# A constrained machine learning surrogate model to predict the distribution of water-in-oil emulsions in electrostatic fields

**DOI:** 10.1038/s41598-024-61535-z

**Published:** 2024-05-15

**Authors:** Ghazal Kooti, Bahram Dabir, Christoph Butscher, Reza Taherdangkoo

**Affiliations:** 1 Department of Petroleum Engineering, Amirkabir University of Technology, Tehran, Iran; 2 Chair of Engineering Geology and Environmental Geotechnics, TU Bergakademie Freiberg, Freiberg, Germany; 3 Department of Chemical Engineering, Amirkabir University of Technology, Tehran, Iran

**Keywords:** Machine learning, Surrogate modelling, XGBoost, Particle size distribution, Water-in-oil emulsions, Inline electrostatic coalescer, Engineering, Chemical engineering

## Abstract

Accurately describing the evolution of water droplet size distribution in crude oil is fundamental for evaluating the water separation efficiency in dehydration systems. Enhancing the separation of an aqueous phase dispersed in a dielectric oil phase, which has a significantly lower dielectric constant than the dispersed phase, can be achieved by increasing the water droplet size through the application of an electrostatic field in the pipeline. Mathematical models, while being accurate, are computationally expensive. Herein, we introduced a constrained machine learning (ML) surrogate model developed based on a population balance model. This model serves as a practical alternative, facilitating fast and accurate predictions. The constrained ML model, utilizing an extreme gradient boosting (XGBoost) algorithm tuned with a genetic algorithm (GA), incorporates the key parameters of the electrostatic dehydration process, including droplet diameter, voltage, crude oil properties, temperature, and residence time as input variables, with the output being the number of water droplets per unit volume. Furthermore, we modified the objective function of the XGBoost algorithm by incorporating two penalty terms to ensure the model’s predictions adhere to physical principles. The constrained model demonstrated accuracy on the test set, with a mean squared error of 0.005 and a coefficient of determination of 0.998. The efficiency of the model was validated through comparison with the experimental data and the results of the population balance mathematical model. The analysis shows that the initial droplet diameter and voltage have the highest influence on the model, which aligns with the observed behaviour in the real-world process.

## Introduction

The measurement of particle size distribution is important in various engineering applications and fundamental research, encompassing a wide range of particles, including droplets, bubbles, and sediments. Predicting particle size distribution is critical for studying the dynamics of multiphase flow^1^. In the field of crude oil production, the presence of impurities within the extracted oil poses various challenges. One common impurity is brine, which can lead to many problems including increased pressure drops in pipelines, catalyst deactivation, fouling and corrosion in equipment, high heat consumption, and low crude oil value^2^. Therefore, the improvement of the dehydration process to reduce the water content in crude oil is essential.

Various techniques have been developed to address this issue, including gravitational, thermal, chemical, mechanical, and electrical coalescence^3–5^. Among these methods, electrostatic coalescence is a widely used approach due to its effectiveness in removing water droplets and its adaptable application across diverse oil compositions and operational conditions. Furthermore, its environmentally friendly approach, requiring fewer chemicals and less heat, aligns with the industry’s growing emphasis on sustainable oil production^6^. This method utilizes electric fields to enhance the collision of water-in-oil emulsions and facilitate the formation of larger droplets, leading to the separation of the dispersed phase from the continuous phase, and minimizing the adverse effects of brine contamination^7^. The classical industrial electrostatic treaters use high-voltage alternating current (AC) or, to a lesser extent, direct current (DC) fields to promote coalescence of a slowly flowing fluid mixture^8,9^. However, these conventional electro-coalescer vessels tend to be large due to extended residence times needed for effective separation.

Recent advancements in electro-coalescence technology, such as inline electrostatic coalescers (IEC), have improved water separation efficiency^10,11^. These devices subject the water/oil mixture to an AC electric field, magnifying droplet sizes and enhancing coalescence rates in the pipeline to facilitate the water separation downstream reducing the reliance on demulsifying chemicals and promoting an environmentally friendly approach^12^. IECs are particularly crucial for increasing the efficiency of dehydration in heavy oil processing and offshore operations, as they effectively counteract the emulsion stabilizing effects of surface-active compounds in heavy crude oil, such as asphaltene and resin while providing a compact design, light weight, and superior performance particularly beneficial in limited-space offshore units.

Previous studies have primarily focused on the modelling of traditional electro-coalescer vessels, taking into account factors such as the strength of the electric field, flow rates, and residence times^13–18^. However, the phenomenon of droplet breakage, which can occur simultaneously and influence separation efficiency, has been largely disregarded. Furthermore, limited attention has been given to modelling inline electrostatic devices. Considering the growing demand to address flow conditioning challenges, particularly in constrained environments like offshore platforms, and the necessity to enhance the efficiency of heavy oil processing, it becomes essential to develop a thorough understanding of IECs. Therefore, in our previous study^11^, we developed a mathematical model using population balance equations (PBE) to consider both coalescence and breakage of emulsion droplets under the influence of a static electric field within an IEC. The model predictions closely matched experimental data, examining factors such as electric field intensity, inlet flow rate, and residence time to understand their impact on droplet size distribution and separation efficiency.

The prediction of electrostatic water separation efficiency, which is based on predicting the temporal size distribution of water-in-oil emulsions requires cumbersome calculations due to the complex interactions of multiphase dynamics, fluid mechanics, and electrostatic forces. Implementing the direct population balance model for these calculations poses challenges in terms of computational efficiency and increased computational cost, making it difficult for scenarios where quick predictions or resource-efficient solutions are required. In response, surrogate machine learning (ML) models can be used as practical alternatives. Machine learning algorithms are inherently data-driven and are capable of identifying meaningful patterns and connections within available data^19,20^. The ML surrogate model captures the essential features and patterns of the original model, enabling faster and more efficient predictions with acceptable accuracy^21^. Surrogate modelling is particularly useful when dealing with complex systems or simulations where direct modelling may be less practical due to computational constraints or resource limitations. There is limited existing research on application of machine learning methods to predict the size distribution of droplets in the crude oil dehydration process. Ranaee et al. conducted a study utilizing artificial intelligence techniques to assess the performance of a traditional crude oil demulsification system. They achieved this by combining global sensitivity analysis, machine learning methods, and rigorous model discrimination criteria^18^. However, to the best of our knowledge, there has been no research conducted on the utilization of machine learning surrogate modelling for inline electrostatic coalescer systems.

In this study, we employed an extreme gradient boosting (XGBoost) model, fine-tuned with a genetic algorithm (GA), to estimate the distribution of droplet sizes across a diverse range of input variables. We used an extensive dataset from our validated mathematical model^10,11^ in a controlled environment. In the next step, two penalty terms were incorporated into the objective function of the XGBoost algorithm to prevent high modelling deviations and eliminate negative outputs. The XGBoost model incorporates critical parameters of the electrostatic dehydration process, including droplet diameter, voltage, crude oil properties, temperature, and residence time as input factors, and predicts the number of water droplets per unit volume as its output. We employed permutation and Shapley additive explanations (SHAP) methods to evaluate the influence of the input parameters on the modelling output. The efficiency of the constrained XGBoost model was assessed by comparing its predictions to a standard XGBoost model as well as experimental data^12,22^ and the outcomes of the phenomenology-based mathematical model^11^.

## Methodology

### Mathematical model

**Figure 1:**
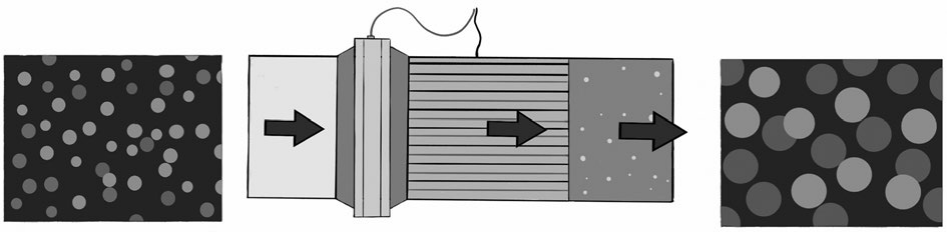
A schematic representation of an inline electrostatic coalescer device^10,12^.

**Figure 2:**
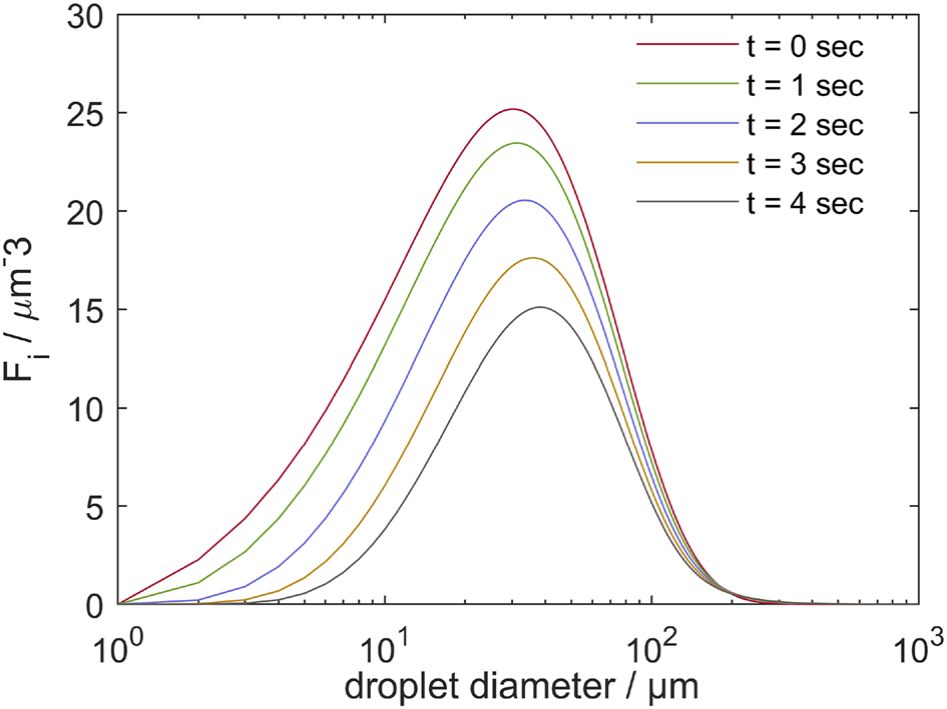
Temporal changes in the number density distribution of water droplets ($F_{i}$) with different diameters per unit volume at the outlet of IEC^11^.

Kooti et al. utilized a population balance modelling approach to simulate the dynamic and complex processes of coalescence and breakage of water-in-oil emulsions in inline electrostatic coalescers^11^. IEC is a pipe-based device equipped with a series of insulated active and grounded electrodes, exposed to an AC electric field^12^ (Fig. 1). As illustrated in Fig. 2, this system enhances the separation of water from crude oil by destabilizing water-in-oil emulsions and promotes a shift in the size distribution towards larger water droplets. The electric field causes dispersed water droplets to become polarized and collide in a moderately turbulent flow regime. The polarized drops are provided with a strong-range attraction force that enables them to break the inter-facial film between them and eventually merge to create a larger droplet^22^.

The Population Balance Equations (PBE) were employed to provide a macroscopic level understanding of how the size distribution of particles changes over time in a liquid-liquid system, assuming that the spatial distribution of droplets was random and homogeneous. The PBE was discretized first in the internal coordinate, representing droplet size, using the method of classes. Subsequently, discretization was applied to the external coordinate, representing time. The closure of the PBE was achieved by developing coalescence and breakage kernels to accurately capture the system’s behavior. The results demonstrate the ability of the model to accurately simulate droplet coalescence and breakage in emulsified oil while predicting droplet size distribution and water removal efficiency. For a more in-depth mathematical background, refer to Kooti et al. in the cited literature^11^.

### Machine learning surrogate model

Machine learning has exhibited significant success in domains where identifying non-linear relationships is often challenging^18,23–27^, while mathematical modelling is based on the underlying physical and chemical processes of the phenomenon^28^, particularly in areas like computational fluid dynamics, where deriving causal connections is very important^29^. To utilize the advantages of both approaches, we developed a surrogate ML model to predict the behaviour of an electrostatic coalescer, leveraging a dataset obtained using our previously published mathematical model^11^. The surrogate model is based on the XGBoost algorithm^30^ coupled with a genetic algorithm for hyperparameters tuning. To ensure the model’s robustness and reliability, we ran the algorithm 500 times and selected the model having the lowest mean squared error (MSE) on the test set as our final GA-XGBoost model. This approach minimizes the impact of random factors and potential inconsistencies.

### Extreme gradient boosting

XGBoost is an advanced supervised algorithm for both classification and regression tasks. Its core principle involves the aggregation of multiple weak predictors, predominantly decision trees, to construct a robust predictive model. XGBoost addresses the common challenge of overfitting associated with tree-based algorithms by sequentially integrating numerous tree models^30,31^. The model expression can be written as follows^30,32,33^:

1 $$\begin{aligned} {{\hat{y}}}_i = \sum _{k=1}^{K} f_k(x_i) \end{aligned}$$

where $f_k$ represents the $k$-th tree model, $y_i$ stands for the predicted value for the sample $x_i$, and the loss objective function for the learning process is defined as:

2 $$\begin{aligned} \textrm{Obj}^{(t)} = \sum _{i=1}^{n} l\left( y_i, \widehat{y}^{(t-1)}_i + f_t(x_i) \right) + \Omega (f_t) + \text {constant} \end{aligned}$$

where $l$ represents the differentiable convex loss function that measures the difference between the prediction $\widehat{y}_i$ and the target ${y}_i$. $\Omega (f_t)$ is the regularization term and can be described as follows:

3 $$\begin{aligned} \Omega (f) = \gamma T + \frac{1}{2}\lambda \Vert \varvec{\omega } \Vert ^2 \end{aligned}$$

where $T$ denotes the number of branches within the decision tree algorithm, and $\omega$ represents the vector of branch parameters, following the second-order expansion of Eq. (2), the revised objective function can be written as:

4 $$\begin{aligned} Obj^{(t)} &\approx \sum _{i=1}^{n} l\Bigg (y_i, {{\hat{y}}}_i^{(t-1)} + g_i f_t(x_i) + \frac{1}{2} h_i f_t^2(x_i)\Bigg ) + \Omega (f_t) + \text {constant} \\g_i &= \delta _{{\hat{y}}_{(t-1)}}l(y_i, {\hat{y}}_i^{(t-1)})\\h_i &= \delta ^2_{{\hat{y}}_{(t-1)}}l(y_i, {\hat{y}}_i^{(t-1)}) \end{aligned}$$

where $g_i$ and $h_i$ represent the initial and subsequent derivatives of the loss function, denoted as *l*, at the value of $y^{(t-1)}$. To prevent overfitting during training, the algorithm does not simultaneously train all regression trees; instead, it sequentially incorporates decision trees. Consequently, when incorporating *t* trees, the prior $t-1$ trees have already undergone training, making $l(y_i, {\hat{y}}^{(t-1)}_i)$ essentially a fixed factor. Eventually, this simplifies the objective function to:

5 $$\begin{aligned} Obj^{(t)} \approx \sum _{i=1}^{n}\Bigg ( g_i f_t(x_i) + \frac{1}{2} h_i f_t^2(x_i)\Bigg ) + \Omega (f_t) + \text {constant} \end{aligned}$$

We incorporated two penalty terms in the objective function of the XGBoost algorithm to obtain more meaningful predictions. The first penalty function, $\text {Penalty}_1$, targeted the residuals between the observed ($y_{\text {obs.}}$) and the machine learning predicted ($y_{\text {pred.}}$) values of population of droplets per unit volume. This function was defined as:

6 $$\begin{aligned} \text {Penalty}_1 = {\left\{ \begin{array}{ll} |y_{\text {obs.}} - y_{\text {pred.}}|^3, &{} \text {if } |y_{\text {obs.}} - y_{\text {pred.}}| > t \\ |y_{\text {obs.}} - y_{\text {pred.}}|, &{} \text {otherwise} \end{array}\right. } \end{aligned}$$

where the threshold $t$ was set at $0.001$, with residuals exceeding this threshold being raised to the power of $3$, the value of $t$ was determined through trial and error in subsequent steps.

Additionally, $\text {Penalty}_2$ was applied to discourage negative predicted values. This function was defined as:

7 $$\begin{aligned} \text {Penalty}_2 = {\left\{ \begin{array}{ll} - c \times y_{\text {pred.}}, &{} \text {if } y_{\text {pred.}} < 0 \\ 0, &{} \text {otherwise} \end{array}\right. } \end{aligned}$$

where the adjusting parameter $\text {c}$ is obtained through an iterative process.

The overall mean squared error was computed as follows:

8 $$\begin{aligned} \text {MSE}_{\text {overall}} = \text {MSE} + \frac{1}{N} \sum _{i=1}^{N} \text {Penalty}_1(y_{\text {obs.},i} - y_{\text {pred.},i}) + \frac{1}{N} \sum _{i=1}^{N} \text {Penalty}_2(y_{\text {pred.},i}) \end{aligned}$$

where *N* is the total number of observations.

### Genetic algorithm

Genetic Algorithm (GA) is an optimization algorithm that mimics the process of natural evolution, where the survival of fitter creatures and their genes were simulated^34^. This algorithm excels in exploring and exploiting the search space through the iterative application of genetic operators to enhance a population of potential solutions^35^. In this study, GA was utilized to tune the hyperparameters of the XGBoost model, such as the number of estimators, maximum depth, learning rate, subsample ratio, and column subsampling ratio.

The GA operates through three main genetic operators: selection, crossover, and mutation^36^. The selection process uses a roulette wheel strategy, where the likelihood of an individual being selected for reproduction is proportional to its fitness. This method ensures that better-performing hyperparameter sets have a higher chance of propagating to subsequent generations^37^. Crossover, specifically a two-point crossover, is then applied to selected individuals. This operator combines parts of two parent solutions to produce new offspring, promoting the mixture of good traits and the discovery of better-performing hyperparameter combinations. The mutation process, described by the following equation, introduces random changes to offspring^38^:

9 $$\begin{aligned} x_{i}^{(t+1)} = {\left\{ \begin{array}{ll} \text {a new value} &{} \text {if } \text {rand()} < \text {mutation rate} \\ x_{i}^{(t)} &{} \text {otherwise} \end{array}\right. } \end{aligned}$$

where $x_{i}^{(t+1)}$ represents the state of the $i-\text {th}$ individual in the population in iteration $t + 1$. The function $\text {rand()}$ generates a random number between 0 and 1, and the mutation rate is a predefined threshold that determines the likelihood of a mutation. This process enables the algorithm to explore new areas in the hyperparameter space, potentially leading to better solutions.

The GA parameters significantly impact the optimization process. Key parameters include population size and maximum iterations, which define the extent and depth of the search; a larger population and more iterations expand the search but require more computational resources. Mutation probability and elite ratio maintain a balance between discovering new solutions and retaining the best ones. Crossover probability and the proportion of parents affect the population’s diversity, with higher crossover probability enhancing diversity and a greater parents’ portion ensuring the persistence of best-performing solutions^39^. These GA parameters were chosen iteratively to achieve a balance between comprehensive exploration and computational efficiency.

### Model development and evaluation

We employed the data obtained from the mathematical model presented by Kooti et al. for the model development^11^. The dataset consists of 13600 data points. Table 1 summarizes the statistical analysis of the dataset, including properties of the fluid and the electrostatic coalescence system.

The compiled dataset encompasses a wide range of characteristics, including the diameter of droplets ($d_{i}$), voltage (V), crude oil density ($\rho$), viscosity ($\mu$), residence time (t), temperature (T), and the number of water droplets with a specific diameter per unit volume ($f_{i}$), which represents the size distribution of the dispersed phase.

**Table 1:** Summarized statistics for electrostatic dehydration system and fluid characteristics.

	$d_{i} / \upmu \hbox {m}$	$V / \hbox {kV}\,\hbox {cm}^{-1}$	$\rho / \hbox {kg}\,\hbox {m}^{-3}$	$\upmu / \hbox {mPa}\,\hbox {s}$	$t / \hbox {s}$	$T / ^{\circ }\hbox {C}$	$f_{i} / \upmu \hbox {m}^{-3}$
Min	1	1	865	13	1	35	0
Max	1000	4	905	38	4	60	25
Mean	500.5	2.5	893.23	30.65	2.97	52.65	1.65
Median	500.5	2.5	905	38	4	60	0.01
Standard deviation	288.74	1.12	18.23	11.39	1.27	11.39	4.66
Kurtosis	− 1.20	− 1.36	− 1.18	− 1.18	− 1.49	− 1.18	10.67
Skewness	− 6.00e−16	− 3.07e−17	− 0.90	− 0.90	− 0.55	− 0.90	3.34

The entire dataset was divided into a training set and a testing set at the split ratio of 4:1. This ratio ensures that a significant amount of data is used for training, while still retaining a robust and representative test set to evaluate model performance. Therefore, following the completion of the hyperparameter tuning phase, the training set was used for model training, while the testing set was used to assess the model’s predictive performance. This step ensures an unbiased evaluation of the model, verifying the model’s ability to generalize to unseen data, and is essential for reducing the risk of over-fitting^20,40^.

The performance of the constrained and standard XGBoost models in predicting droplet population per volume was assessed through standard metrics, including mean squared error (MSE), root mean squared error (RMSE), and the coefficient of determination ($R^2$). We performed residuals analysis, i.e. the difference between predicted values of mathematical and ML model, and comparative analysis to further evaluate the effectiveness of the modelling approach. The relative importance of input parameters was determined by calculating Shapley additive explanations (SHAP) values^41^ and permutation feature importance^42^.

## Results

### Performance analysis

We first ran multiple simulations to determine the optimal bounds of hyperparameters of the XGBoost model and to fine-tune the user-defined parameters in the genetic algorithm. Following this, we chose the hyperparameter boundaries as detailed in Table 2 for building the models. The maximum number of iterations was set to 30, with a population size of 5 individuals. The mutation probability was 0.12, and elitism was applied to the top 2% of the population. Crossover was performed with a probability of 0.9 using a two-point method, and 5% of the population was selected as parents. The selection process employed the ’roulette’ method, and mutations were applied randomly. There was no specified limit for the maximum number of iterations without improvement. The parameter $\text {c}$ in the equation (7) was determined iteratively to be 100.

**Table 2:** Comparison of hyperparameter optimal values for GA-XGBoost and constrained GA-XGBoost models.

Hyperparameter		Optimum value	
Name	Bound	GA-XGBoost	Constrained GA-XGBoost
Learning rate (LR)	0.1–0.9	0.559	0.116
Max depth (MD)	1–20	17	15
Number of estimators (NoE)	50–500	78	477
Subsample	0.1–0.9	0.369	0.426
Colsample Bytree	0.1–0.9	0.598	0.896

**Figure 3:**
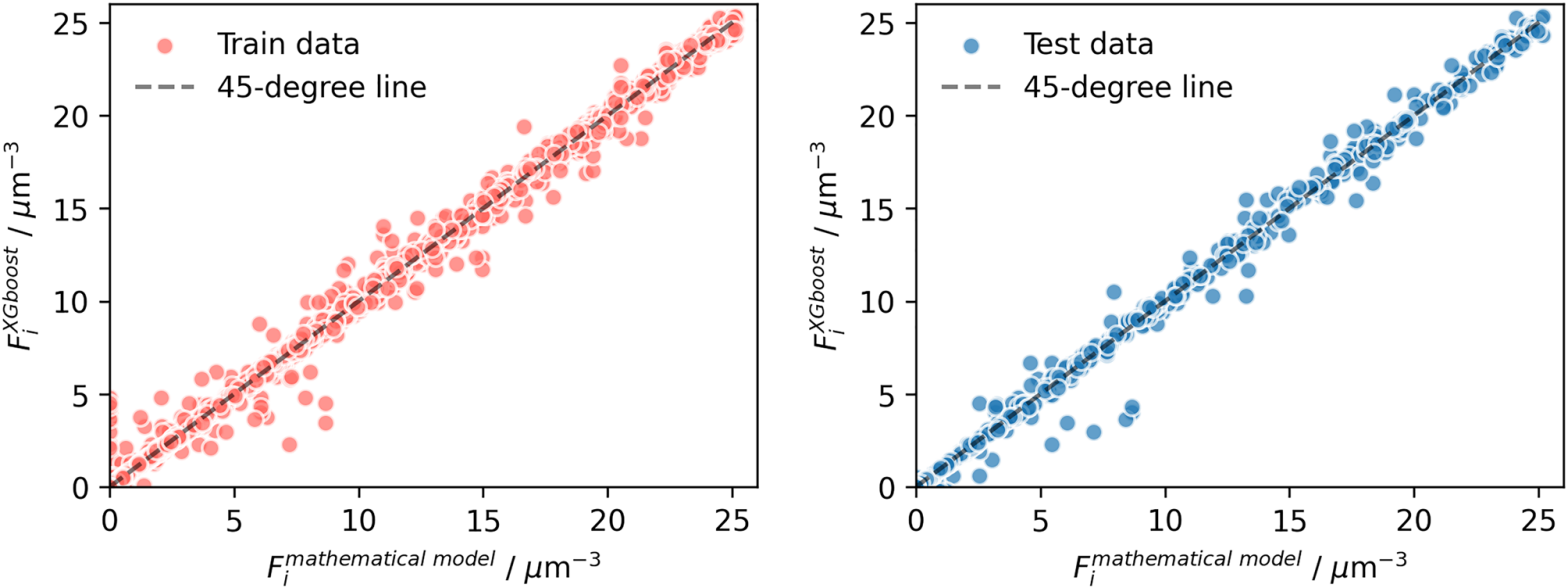
Regression plots comparing the standard GA-XGBoost model predictions to mathematical model data for the droplet population per unit volume.

**Figure 4:**
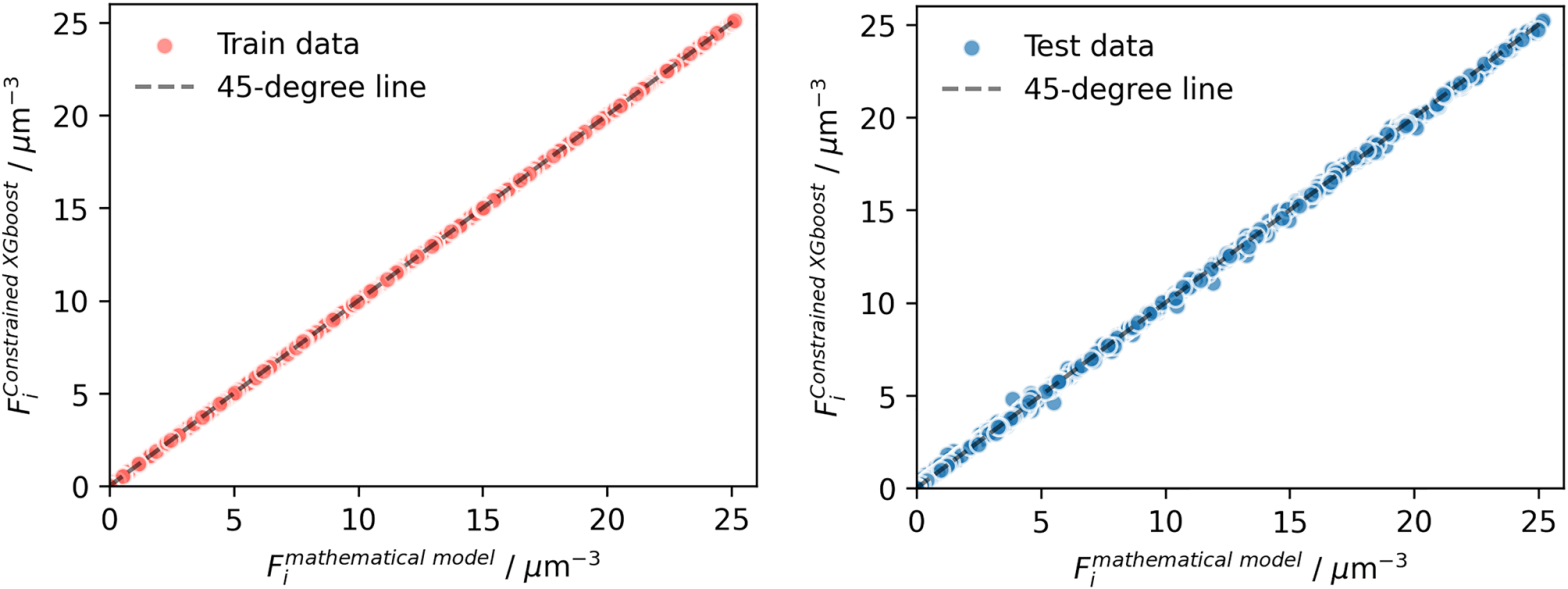
Regression plots comparing the constrained XGBoost model predictions to mathematical model data for the droplet population per unit volume.

The train and test set performance values for the two models, standard GA-XGBoost and Constrained GA-XGBoost, were compared in Table 3. For the standard GA-XGBoost, the MSE, the RMSE, and the $R^2$ were 0.106, 0.325, and 0.995. In contrast, the constrained GA-XGBoost model demonstrated superior performance with a MSE of 0.005, a RMSE of 0.069, and a $R^2$ value of 0.998, respectively. These performance metrics indicate that the constrained GA-XGBoost model exhibits better predictive accuracy on the test set across all metrics compared to the standard GA-XGBoost model. In addition to the performance metrics, the regression plots in Figs. 3 and 4 clearly illustrate a stronger correlation between the predicted and observed droplet population per unit volume in constrained GA-XGBoost model, showing robust predictive capabilities on previously unseen data, confirming its capability to obtain the fundamental dataset pattern.

**Table 3:** Comparison of training and testing performance metrics for GA-XGBoost models.

Model	MSE		RMSE		$R^2$	
	Train	Test	Train	Test	Train	Test
Standard GA-XGBoost	0.0502	0.106	0.224	0.325	0.997	0.995
Constrained GA-XGBoost	3.9e−06	0.005	0.002	0.069	0.999	0.998

**Figure 5:**
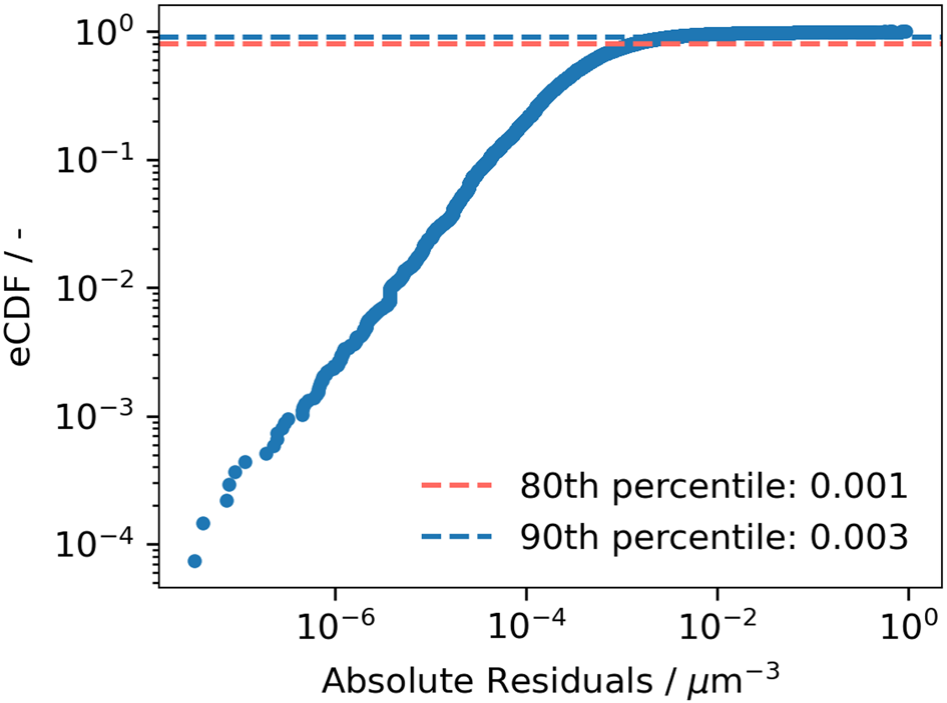
Empirical cumulative distribution function (eCDF) of absolute residuals of the constrained XGBoost model.

The empirical cumulative distribution function (eCDF) of absolute residuals was investigated for evaluating the performance of the ML model. Residuals quantify the difference between observed simulation outputs and the corresponding predictions generated by the model. The eCDF plot depicts the fraction of data points where the absolute residuals fall below a specific threshold on the x-axis. As illustrated in Fig. 5, plotted on logarithmic scales, a steep incline in the curve at lower residuals indicates a significant concentration of data points with prediction errors close to zero. Additionally, the 80th and 90th percentiles of the absolute residuals stand at 0.001 and 0.003 $\upmu \hbox {m}^{-3}$, respectively, indicating the distribution of errors within the dataset. Percentiles represent specific points in a dataset below which a certain percentage of the observations fall and serve as valuable metrics for understanding the spread and magnitude of errors.

**Figure 6:**
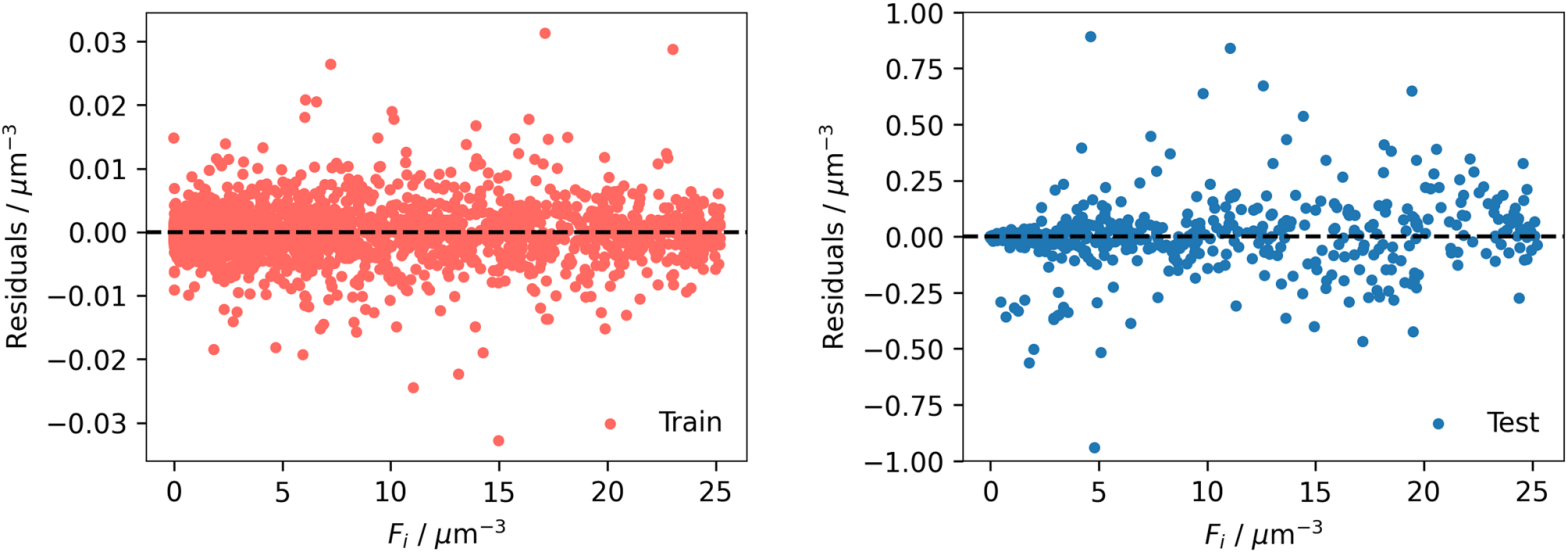
Residual plots of the droplet population per unit volume ($F_{i}$) predicted by the constrained XGBoost model for training and testing data.

**Figure 7:**
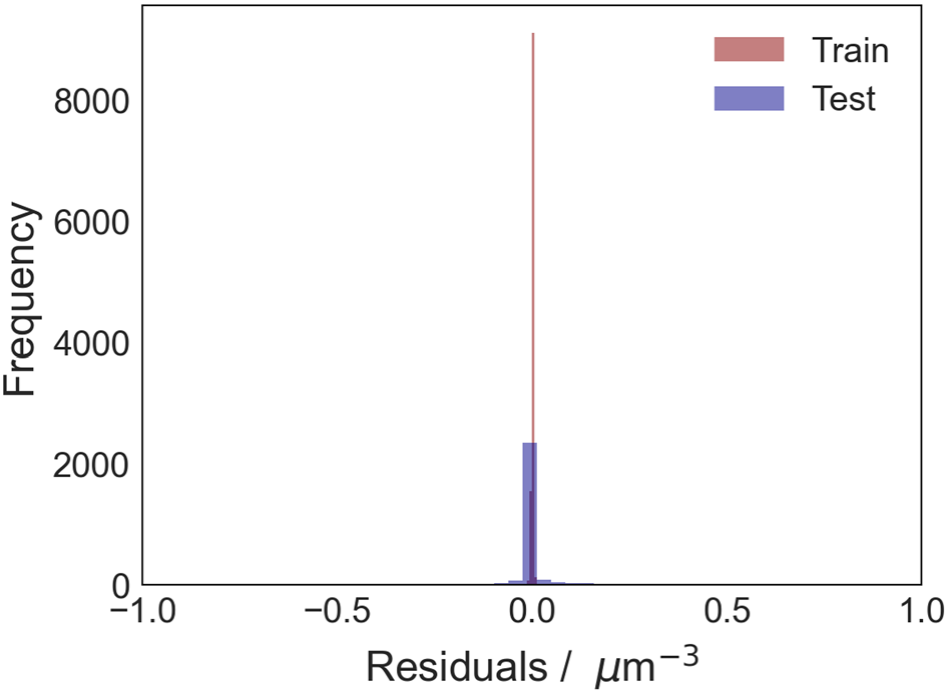
Frequency of residuals in predicting the droplet population per unit volume ($F_{i}$) for training and testing data.

Given the better performance of the constrained GA-XGBoost model, it was utilized for further analysis. As shown in Fig. 6, 98.05% of the residual values, out of a total of 13,600 data points, fall within the range of − 0.25 to 0.25 $\upmu \hbox {m}^{-3}$. This indicates that predictions made by the constrained GA-XGBoost model lie very close to the values of the mathematical model. Furthermore, the residual analysis in Figs. 6 and 7 revealed that random errors were present across the entire spectrum of $F_{i}$ and no systematic bias was detected. This suggests that the model predictions are not systematically over/under estimated within the entire range. This observation further confirms the reliability of the model.

### Feature importance

**Figure 8:**
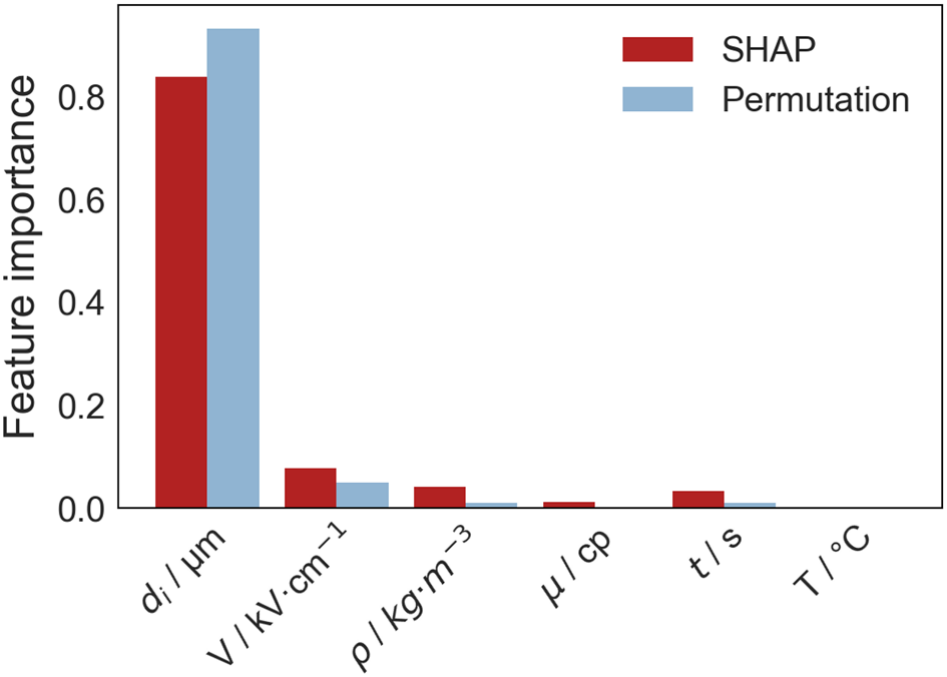
Comparing the relative importance of all the features using SHAP and permutation methods: initial droplet diameter ($\upmu$m), voltage (kV/cm), density (kg/m^3^), viscosity (cp), time (s), and temperature (^∘^C).

We utilized the Shapley Additive Explanations (SHAP) and Permutation techniques to assess the relative importance of input parameters in predicting water droplet size distribution. In the permutation method, each feature was subjected to 30 random permutations. The analysis showed that both methods identified similar rankings in terms of importance (Fig. 8). The diameter of droplets emerged as the most influential factor in determining droplet population per unit volume, representing the size distribution of droplets. The other parameters were ranked in descending order of importance as follows: voltage, crude oil density, residence time, crude oil viscosity, and the temperature of the mixture. Temperature was identified as the least influential feature for two main reasons. First, the narrow range and limited variability of the temperature in the dataset (Table 1) resulted in a smaller impact on the target variable. Second, the unique nature of an inline electrostatic coalescer, in contrast to traditional electrostatic vessels, may be less influenced by temperature due to its significantly shorter residence time. This implies that temperature plays a lesser role in influencing outcomes compared to the other features.

### Comparative analysis

**Figure 9:**
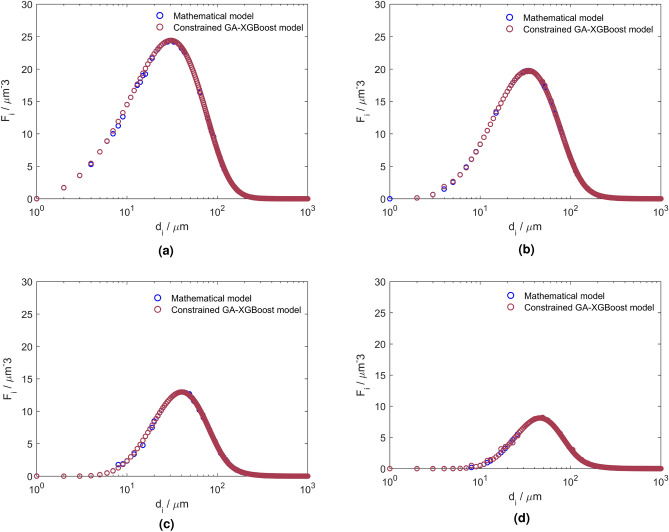
Comparison of droplet population per unit volume predicted by the mathematical and the constrained GA-XGBoost models for varying droplet diameters under four voltage conditions: (**a**) 1 kv/cm, (**b**) 2 kv/cm, (**c**) 3 kv/cm, (**d**) 4 kv/cm.

The comparison between the predicted outputs in the mathematical^11^ and constrained GA-XGBoost model is illustrated in Fig. 9. These plots show results of four different voltages over a wide range of droplet diameters (1–1000 $\upmu \hbox {m}$) while other parameters are kept constant. The selection of varying parameters in this analysis is based on the previously mentioned examination of parameter importance. Consequently, the base parameters for comparison are the two most influential parameters of the dehydration system: droplet diameter and voltage.

The comparison analysis confirms the accuracy of the developed ML model in predicting the droplet size distribution within an IEC system. The plots follow a normal distribution, showing a characteristic bell-shaped pattern with a peak at the centre and a gradual decline in droplet counts towards the outer edges. This pattern implies a tendency for specific droplet sizes to become more prevalent, while less frequent sizes occur towards the extremes of the distribution. Because the coalescence of droplets is the dominant mechanism compared to the breakage of droplets, the process leads to the merging of smaller water droplets into larger ones, which is evident from the reduction in the peaks of the droplet population per unit volume and the reduction of the overall number of droplets.

**Figure 10:**
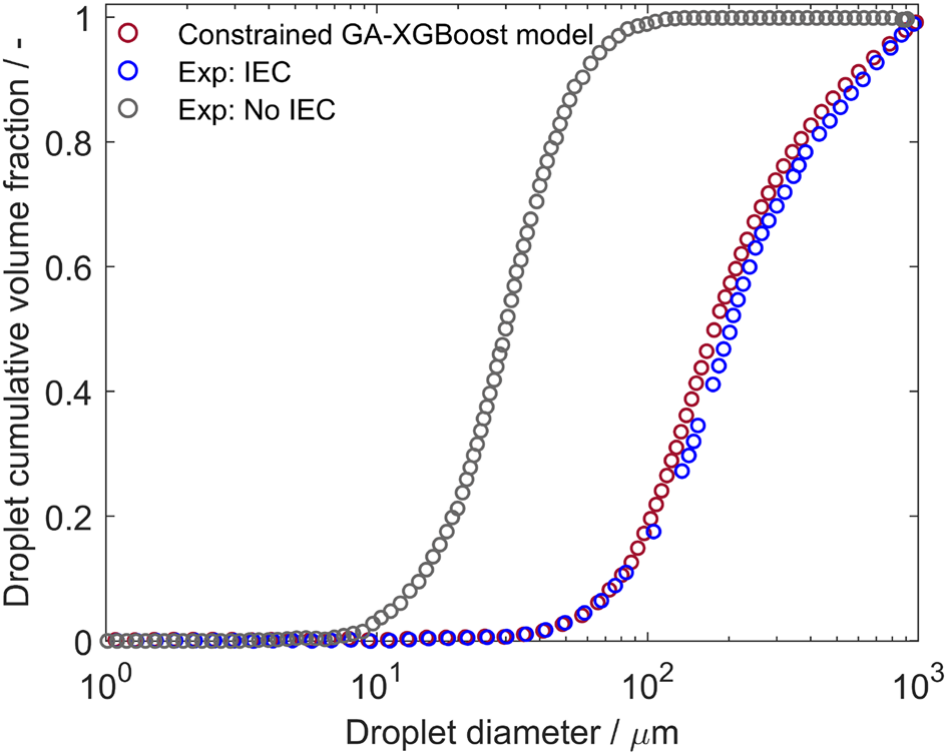
Comparing experimental values of droplet cumulative volume fraction to the constrained GA-XGBoost predicted values.

Figure 10 illustrates the size distribution of droplets in two scenarios: (i) without the utilization of an electric field (IEC switched off) and (ii) with an electric field (IEC switched on), comparing the predictions of the surrogate model to the experimental data^12^. The distribution is represented by plotting the cumulative volume fraction of droplets against their diameter at the electrostatic coalescer outlet. Both scenarios exhibit smooth curves without sudden jumps, indicating a uniform size distribution of droplets. This means that droplets are distributed relatively evenly without significant clustering or localized variations. In the scenario without the presence of an electric field, the analysis of the cumulative volume fraction reveals a distinctive trend. Initially, as the droplet size increases from 10 $\upmu \hbox {m}$, the cumulative volume fraction sharply increases until reaching approximately 50 $\upmu \hbox {m}$, where it approaches a plateau at the value equal to one. This indicates that all of the droplets in this size range have been taken into account, and there are no further larger droplets present in the system.

Another important parameter to analyze is the droplet diameter equivalent to the volume fraction of 0.5, which represents the median droplet size of the system. Analysing this value is essential as it provides a direct comparison of droplet sizes between different scenarios. The median droplet size for the scenario without IEC is approximately 30 $\upmu \hbox {m}$. This indicates that 50% of the dispersed phase volume consists of droplets smaller than or equal to 30 $\upmu \hbox {m}$ in diameter. When comparing the two scenarios, a noticeable change in the droplet diameter range is evident. In the electric field scenario, the cumulative volume fraction curve begins to rise at around 30 $\upmu \hbox {m}$ and reaches one at the droplet diameter of 1000 $\upmu \hbox {m}$, with the median droplet size value equal to approximately 300 $\upmu \hbox {m}$. This shift indicates a considerable change in the droplet size distribution, emphasizing the dominance of larger droplets in the scenario with the electric field, compared to the scenario without it. Therefore, we conclude that the presence of the electric field has a substantial impact on the distribution of droplet sizes and consequently enhances the water removal process. It is evident that the cumulative droplet size distribution obtained by the constrained GA-XGBoost model is consistent with the experimental results.

## Discussion

Coupling XGBoost with Genetic Algorithms for hyperparameter optimization resulted in a robust surrogate model for predicting the behaviour of dispersed droplets in IEC. The GA navigated a high-dimensional search space efficiently, optimizing the hyperparameters. The analysis of feature importance through SHAP and permutation techniques ranked the influence of each input feature on model predictions. The identification of the initial diameter of droplets as the most influential factor aligns with empirical evidence^12^, confirming the model’s ability to capture meaningful relationships. Moreover, the absence of systematic bias, illustrated in Figs. 6 and 7, is important for the applicability of the model in practical scenarios.

While prior studies have primarily focused on conventional electro-coalescence vessels and neglected droplet breakage, our study addresses these gaps and provides more realistic predictions of droplet behaviour in an inline electrostatic field. The significance of this study is the pioneering application of machine learning to the domain of inline electrostatic coalescence. This novel approach addresses the critical need in the crude oil production industry to increase the efficiency of the dehydration process, particularly in constrained environments like offshore platforms, and also for processing heavy oil mainly because it contains natural emulsifiers that cause the water-in-oil emulsions to become more stable which consequently results in low efficiency of electrostatic coalescence^43^.

Our model efficiently predicts the temporal size distribution of water-in-oil emulsions and consequently provides a cost-effective and reliable means to enhance separation efficiency by optimizing the process design and operational conditions. Moreover, this model has positive environmental outcomes, specifically by diminishing the reliance on de-emulsifying chemicals in the crude oil dehydration process. Currently, the industry heavily depends on these chemicals to destabilize the interface between oil and water and facilitate water separation. However, improving the water removal efficiency using our model results in a reduced need for de-emulsifying chemicals, contributing to a more sustainable and eco-friendly approach by lowering the environmental impact associated with the production, usage, and disposal of these chemicals. Therefore, the developed model aligns with the global momentum toward greener practices in the crude oil production sector, emphasizing its positive role in fostering environmental responsibility.

However, it is essential to acknowledge the performance of ML models heavily relies on the quality and representatives of the training data. It is essential to highlight characteristics of the electrostatic device that were not explored in our study, such as different electrode configurations, due to the unavailability of relevant experimental data in the literature, and consequently in the mathematical model. Future investigations can focus on expanding the dataset to include a broader array of diverse industrial settings and also investigating the combination of machine learning with different mathematical models. On the other hand, based on the results of the feature importance analysis, it will also be valuable to explore the impact of reducing input features by excluding the least influential ones. This could potentially simplify the model while maintaining its predictive accuracy. Future research could delve into a comparative analysis, assessing the changes in model accuracy when using a reduced set of input features compared to our current model that incorporates six inputs to offer insights into an optimal configuration of input features.

## Summary and conclusion

In this study, we developed a constrained machine learning surrogate model for predicting the size distribution of water-in-oil emulsions in inline electrostatic coalescers (IECs) as a practical alternative to a mathematical model based on population balance equations to facilitate fast and accurate predictions. This model is valuable in addressing challenges encountered in crude oil dehydration processing, including equipment corrosion and catalyst deactivation. The compact and lightweight design of IEC not only addresses spatial constraints for offshore operations but also demonstrates high efficiency in processing heavy oil with stable emulsions, reducing reliance on demulsifying chemicals and contributing to a more environmentally friendly approach.

We employed an XGBoost algorithm with hyperparameter optimization using a genetic algorithm. We incorporated two penalty terms into the objective function of the algorithm to enhance the physical interpretability and accuracy of our model compared to the standard GA-XGBoost model. These terms discouraged high modeling deviations and negative predictions. The results of the test set revealed the precision of the constrained GA-XGBoost model with MSE of 0.005 and an $R^2$ value of 0.998. The comparative analysis further confirmed the accuracy of the constrained ML model when compared to the experimental data and the outcomes of the mathematical model. Residual analyses showed the model’s reliability, detecting no systematic bias and revealing a majority of residuals tightly clustered around zero. Furthermore, we used SHAP and Permutation methods to assess the importance of the six input features, and results showed the initial droplet diameter and the electric field voltage were the most influential parameters. In conclusion, the surrogate machine learning model provides a practical alternative for accurately describing the evolution of droplet size distribution in inline electrostatic coalescers, serving as a valuable tool to enhance the performance of the dehydration process and advance its practical applications in the petroleum industry.

## Data Availability

The datasets generated during and/or analysed during the current study are available from the corresponding author on reasonable request.
