# Author Correction: A constrained machine learning surrogate model to predict the distribution of water-in-oil emulsions in electrostatic fields

**DOI:** 10.1038/s41598-024-68240-x

**Published:** 2024-07-24

**Authors:** Ghazal Kooti, Bahram Dabir, Christoph Butscher, Reza Taherdangkoo

**Affiliations:** 1 Department of Petroleum Engineering, Amirkabir University of Technology, Tehran, Iran; 2 Chair of Engineering Geology and Environmental Geotechnics, TU Bergakademie Freiberg, Freiberg, Germany; 3 Department of Chemical Engineering, Amirkabir University of Technology, Tehran, Iran

Correction to: *Scientifc Reports* 10.1038/s41598-024-61535-z, published online 15 May 2024

In the original version of this Article Ghazal Kooti was incorrectly affiliated with ‘Chair of Engineering Geology and Environmental Geotechnics, TU Bergakademie Freiberg, Freiberg, Germany’. The correct affiliation is listed below:

Department of Petroleum Engineering, Amirkabir University of Technology, Tehran, Iran.

The original Article has been corrected.

